# Comparison of Ultra-Magnifying Endocytoscopic and Hematoxylin-Eosin-Stained Images of Lung Specimens

**DOI:** 10.3390/diagnostics13051003

**Published:** 2023-03-06

**Authors:** Misato Kobayashi, Noriaki Kurimoto, Ryosuke Tanino, Yohei Shiratsuki, Takae Okuno, Mika Nakao, Takamasa Hotta, Yukari Tsubata, Makoto Nagasaki, Takashi Nishisaka, Takeshi Isobe

**Affiliations:** 1 Division of Medical Oncology & Respiratory Medicine, Department of Internal Medicine, Faculty of Medicine, Shimane University, Izumo 693-8501, Japan; 2 Division of Pathology, National Hospital Organization Hamada Medical Center, Hamada 697-8511, Japan; 3 Department of Pathology and Laboratory Medicine, Hiroshima Prefectural Hospital, Hiroshima 734-8530, Japan

**Keywords:** endocytoscopy, nuclear feature, peripheral pulmonary lesion, lung cancer

## Abstract

Endocytoscopy enables real-time observation of lesions at ultra-magnification. In the gastrointestinal and respiratory fields, endocytoscopic images are similar to hematoxylin-eosin-stained images. This study aimed to compare the nuclear features of pulmonary lesions in endocytoscopic and hematoxylin-eosin-stained images. We performed an endocytoscopy to observe resected specimens of normal lung tissue and lesions. Nuclear features were extracted using ImageJ. We analyzed five nuclear features: nuclear number per area, mean nucleus area, median circularity, coefficient of variation of roundness, and median Voronoi area. We conducted dimensionality reduction analyses for these features, followed by assessments of the inter-observer agreement among two pathologists and two pulmonologists to evaluate endocytoscopic videos. We analyzed the nuclear features of hematoxylin-eosin-stained and endocytoscopic images from 40 and 33 cases, respectively. Endocytoscopic and hematoxylin-eosin-stained images displayed a similar tendency for each feature, despite there being no correlation. Conversely, the dimensionality reduction analyses demonstrated similar distributions of normal lung and malignant clusters in both images, thus differentiating between the clusters. The diagnostic accuracy of the pathologists was 58.3% and 52.8% (κ-value 0.38, fair), and that of the pulmonologists was 50% and 47.2% (κ-value 0.33, fair). The five nuclear features of pulmonary lesions were similar in the endocytoscopic and hematoxylin-eosin-stained images.

## 1. Introduction

Lung cancer is a leading cause of death in Japan. Identifying driver variants is essential for selecting appropriate targeted therapy for lung cancer [1,2,3]. Bronchoscopy facilitates pathological diagnosis and detection of oncogenes in biopsy specimens [4,5]. Endobronchial ultrasonography is performed in the clinical setting to determine the appropriate biopsy site in peripheral lesions. However, this site often cannot be directly observed during a biopsy, resulting in uncertainty concerning the presence of sufficient tissue for a pathological diagnosis for detecting oncogenes from the target lesion. Endocytoscopy (ECS) enables the observation of a lesion at high magnification (approximately 450×) on a monitor by placing the objective lens in direct contact with the target lesion, with a depth of field of approximately 30 μm from the surface. ECS has been used in the gastrointestinal field in clinical practice, particularly for esophageal, stomach, and colon lesions, with methylene blue staining [6,7,8,9] or crystal violet and methylene blue double staining [7,8]. ECS demonstrates a diagnosis rate comparable to that of biopsy for colorectal lesions, with a high level of diagnostic sensitivity and specificity [7]. Endocytoscopic images of colorectal lesions are similar to their hematoxylin-eosin (H&E)-stained images [7,8], and colorectal endocytoscopic classification is used for the diagnosis [8]. Nevertheless, some respiratory field studies have reported endocytoscopic observations [10,11,12,13,14,15], and no quantitative studies have compared the endocytoscopic findings with the pathologic findings. This study aimed to obtain the endocytoscopic findings of pulmonary lesions for quantitative comparison of the nuclear features with those of H&E-stained images. We also intended to examine the inter-observer agreement of endocytoscopic videos for investigating the diagnostic ability of ECS.

## 2. Materials and Methods

### 2.1. Study Design

This prospective, single-center, observational study was performed at the Shimane University Hospital. We conducted this study from September 2019 to September 2021 in 40 patients who underwent surgical resection of pulmonary lesions at the Department of Cardiothoracic Surgery, Division of Thoracic Surgery, Shimane University. Patients aged 20 years or older who underwent surgical resection of an intrathoracic nodule or mass and gave written informed consent were included. The exclusion criterion was any patient considered inappropriate at the discretion of the doctor in charge. The target sample size was 50 participants. The enrolment period was from October 2019 to March 2020. We analyzed the data until September 2021. The study was approved by the Medical Research Ethics Committee of the Shimane University Faculty of Medicine (approval number: 4020) and registered in the UMIN Clinical Trials Registry (UMIN 000038136). Informed consent was obtained from all patients before lung resection. The study was conducted in accordance with the tenets of the Declaration of Helsinki.

### 2.2. Endocytoscopy

**Figure 1:**
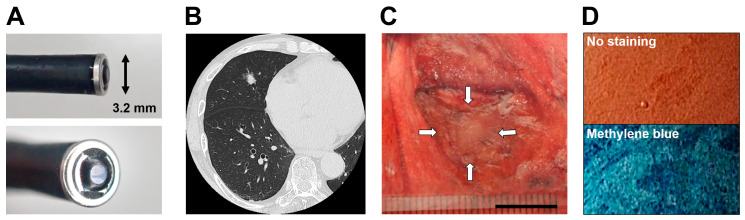
Endocytoscopy (ECS) overview for resected lung specimens. (**A**) Appearance of an endocytoscope (Source: courtesy of Olympus Medical Systems). (**B**) High-resolution computed tomography of a patient with adenocarcinoma. (**C**) Macroscopic appearance of a lesion (white arrows: adenocarcinoma) resected from the patient. Scale bar: 10 mm. (**D**) Endocytoscopic images of the lesion with and without methylene blue staining.

We used a catheter-type endocytoscope (prototype XEC-300-2, Olympus Medical Systems, Tokyo, Japan) with a length of 380 cm and an outer diameter of 3.2 mm at the tip. An endocytoscopic image can provide an observation field, an observation depth, and a horizontal resolution of 300 × 300 μm, 0–30 μm, and 4.2 μm, respectively, with 450-fold magnification on a 14-inch monitor (Figure 1A).

### 2.3. Acquisition of the Endocytoscopic Images

Computed tomography (CT) images showed the location of a target lesion (Figure 1B). Thoracic surgeons performed surgical resection of the lesion. A pathologist cut into the center of the lesion in the resected specimens. In cases that required rapid intraoperative diagnosis, the part that remained following the removal of a portion of the resected tissues for frozen sections by the pathologist was used for endocytoscopic observation (Figure 1C). Without staining, no relevant findings regarding the lesion were revealed. Methylene blue staining revealed dark-blue cell nuclei at the same lesion site (Figure 1D). Therefore, we stained the cut surface with a drop of 0.25–0.5% methylene blue using a 1-mL syringe with a 23-G needle. The tip of the endocytoscope was placed directly on the cut surface, and we immediately observed the stained lesion on the monitor screen. Additional drops of methylene blue were added for large lesions as necessary. We attempted to observe the normal lung area in a similar manner. These observations were recorded using a video recorder (IMH-20; Olympus Medical Systems). The procedure was completed within 3 h of the resection. The specimens were fixed with formalin and stained with H&E.

### 2.4. Analyzing Nuclear Features

#### 2.4.1. Nuclear Extraction

ImageJ 1.53o (National Institutes of Health, Bethesda, MD, USA) was used for image quantification. We obtained H&E-stained images of the pathologic tissues, and every image was processed using the ImageJ “subtract background” feature (parameters: rolling ball radius = 20, light background; the other functions were unused). Endocytoscopic images of the normal lung tissue and lesions were obtained from the endocytoscopic observation videos using Edius 8 Pro version 8.53 (Grass Valley, Montreal, Canada). Every image was cropped at the observation area (x = 780, y = 188, w = 660, and h = 680) using ImageJ. Dark endocytoscopic images (mean gray value < 50) were excluded owing to the difficulty in nuclear extraction. We extracted the stained nuclear component using Colour Deconvolution 1.7 plugin. The H&E (Colour_1) and Masson Trichrome (Colour_3) vectors were used for the H&E-stained and endocytoscopic images, respectively. We performed subsequent processing in the following order: median (parameter: radius = 3), subtract background (parameters: rolling ball radius = 20, light background; the other functions were unused), auto local threshold (parameters: method = Phansalkar, radius = 20 [H&E images] or 10 [ECS images], parameter_1 = 0, parameter_2 = 0, white objects on black background), despeckle, and watershed (parameters: iterations = 1, count = 1, EDM output = overwrite; the other functions were unused). Moreover, we obtained Voronoi diagram images using Voronoi processing (with the same parameters as those used for the watershed process) and a nuclear-extracted image.

#### 2.4.2. Quantification of the Nuclear Features

To obtain the nuclear features, we performed an Analyze Particles (parameters: size = 0 to infinity, circularity = 0.00–1.00) command using nuclear-extracted images (nucleus area, nuclear number, circularity [(4π × area)/(perimeter^2^)], roundness [(4 × area)/(π × major axis^2^)]) and Voronoi diagram images (Voronoi area). Moreover, we calculated the nuclear number per area, mean nucleus area (the sum of the nucleus area/nuclear number), median circularity, coefficient of variation (CV) of roundness, and median Voronoi area of each image.

#### 2.4.3. Two-Dimensional Projection of Five Nuclear Features

Min-max normalized values of the nuclear features were calculated using the following formula: normalized values = (value − minimum value)/(maximum value − minimum value). The distinct nuclear trajectory was two-dimensionally mapped using principal component analysis (PCA) and nonlinear uniform manifold approximation and projection (UMAP) [16] to reduce the dimension from the normalized values of the nuclear number per area, mean nucleus area, median circularity, CV of roundness, and median Voronoi area.

### 2.5. Endocytoscopic Observation and Inter-Observer Agreement

Two pathologists and two pulmonologists reviewed the recorded videos of the endocytoscopic observation and evaluated the lesions. The observers were requested to classify malignancy upon identifying unequal nuclei size and uneven distribution of the nuclei in the video. When none or one of the two findings was present, the lesion was classified benign by endocytoscopic (“ECS-benign”). On identifying a lesion as malignant, the observers were requested to differentiate it as lung adenocarcinoma by ECS (“ECS-adeno”) or another malignancy by ECS (“ECS-non-adeno”), according to the following criteria. ECS-adeno showed enlarged and irregularly sized nuclei piled up on the alveolar wall. Conversely, ECS-non-adeno was classified as malignancy, except adenocarcinoma, for unevenly distributed enlarged and irregularly sized nuclei, without piling up on the alveolar wall. Accuracy (acc) was calculated using the following formula: acc = (number of classified cases matched with pathology)/(number of cases). Inter-observer agreement was determined using Kappa statistics. All of the observers were blinded to the information regarding the patients who participated in the endocytoscopic observation.

### 2.6. Statistical Analyses

We performed the Kruskal–Wallis test with Dunn’s multiple comparison test and a simple linear regression using GraphPad Prism 9.3.1 (GraphPad, San Diego, CA, USA). The results were considered statistically significant at *p* < 0.05. Correlations between the nuclear features of the endocytoscopic and H&E-stained images were calculated using the Pearson correlation coefficient. Kappa statistics were calculated using JMP Pro 14.2.0 (SAS Institute, Cary, NC, USA). Agreements between observers were defined based on κ values as follows: poor, κ < 0; slight, κ = 0–0.20; fair, κ = 0.21–0.40; moderate, κ = 0.41–0.60; substantial, κ = 0.61–0.80; and almost perfect, κ = 0.81–1.00.

## 3. Results

### 3.1. Cases and Their Diagnosis

**Figure 2:**
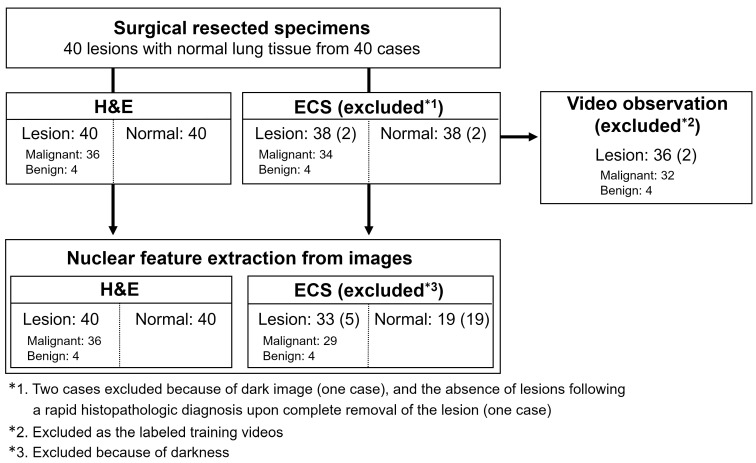
Study overview. All 40 cases were included in the analysis of the H&E-stained images. Thirty-eight cases provided endocytoscopic images. Thirty-three lesions (malignant = 29, benign = 4) and 19 normal lung tissues were included in the nuclear feature analysis of the endocytoscopic images after excluding endocytoscopic images that were too dark to extract the nucleus using ImageJ. In endocytoscopic observation, 36 cases were used to assess inter-observer agreement, excluding two cases as the labeled training videos.

In total, 40 lesions from 40 cases were diagnosed histopathologically (Figure 2). Of the 40 lesions, 36 were malignant (20 lung adenocarcinomas, five squamous cell carcinomas, 4 cases of other types of lung cancer [two large-cell neuroendocrine carcinomas, one carcinoid, and one pleomorphic carcinoma], six metastatic lung tumors [five colorectal cancers and one pancreatic cancer], and one diffuse large B-cell lymphoma). The four benign cases consisted of two granulomas, one lung cyst, and one intrapulmonary lymph node. Thirty-eight lesions and 38 normal lung tissue specimens were observed by ECS (Figure 2). No patient had a history of anticancer therapy or chest radiation therapy.

### 3.2. ECS and H&E Findings of Representatives

**Figure 3:**
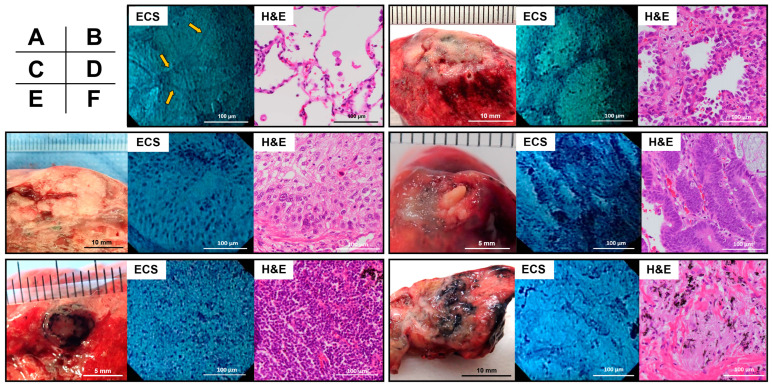
Macroscopic appearance, endocytoscopic images, and hematoxylin-eosin (H&E)-stained images of the resected lesions. (**A**) Normal lung tissue (yellow arrows: alveolar septa). (**B**) Adenocarcinoma. (**C**) Squamous cell carcinoma. (**D**) Lung metastasis from rectal cancer. (**E**) Intrapulmonary lymph node. (**F**) Necrotizing granuloma.

Next, we compared endocytoscopic and H&E-stained images for each specimen. ECS displayed alveolar cell nuclei and slightly stained alveolar septa in an endocytoscopic image of normal lung tissue, similar to that of the H&E-stained image (Figure 3A). Figure 3B depicts an adenocarcinoma. The nodule was 18 × 10 mm in size. The ECS displayed enlarged and irregularly shaped nuclei piled up on the alveolar walls. For the same case, H&E staining displayed cells with a high nuclear-cytoplasmic ratio. Moreover, large nuclei increased along the alveolar walls, thus corresponding to lung adenocarcinoma. Figure 3C depicts a squamous cell carcinoma. The nodule was 20 × 15 mm in size. ECS revealed enlarged, irregularly shaped nuclei forming nests and partially overlapping. H&E staining revealed tumor cells forming nests, thereby corresponding to squamous cell carcinoma. Figure 3D depicts a case of rectal cancer metastasis. The nodule comprised a whitish solid part and a yellowish part, with a total size of 8 × 6 mm. ECS revealed an increase in the number of palisading nuclei. H&E-stained tumor cells were lined with irregularly shaped nuclei and formed atypical gland ducts, which correspond to the metastasis of rectal cancer. Figure 3E depicts a round and black intrapulmonary lymph node measuring 7 × 7 mm in size. ECS revealed clusters of small round nuclei. H&E staining displayed small lymphocytes, which correspond to the intrapulmonary lymph nodes. Figure 3F depicts a case of necrotizing granuloma. The nodule was whitish, soft, and 15 × 9 mm in size. ECS revealed few nuclei with fibrotic fibers, whereas H&E staining revealed proliferated macrophages, fibroblasts, and collagen fibers, thus corresponding to necrotizing granuloma. The aforementioned endocytoscopic findings were similar to those of H&E-stained imaging.

### 3.3. Comparison of the Nuclear Features of the Normal Lung, Benign, and Malignant Lesions

**Figure 4:**
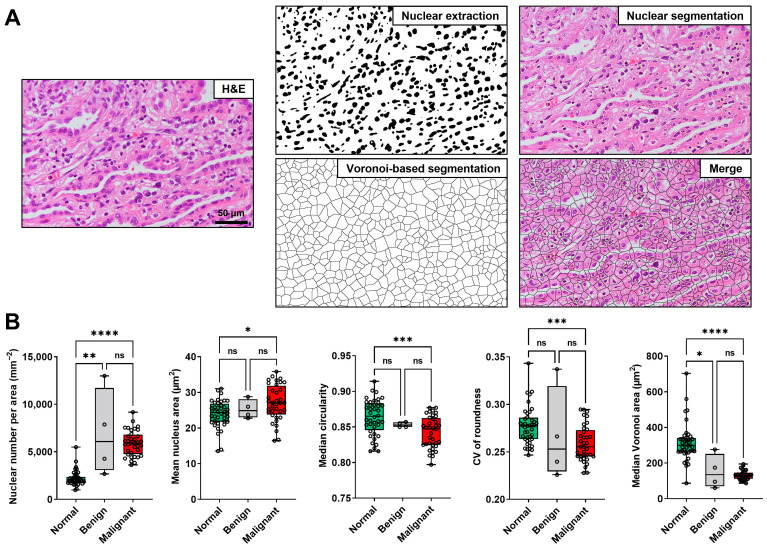
Nuclear feature extraction from the hematoxylin-eosin (H&E)-stained images of the formalin-fixed, paraffin-embedded resected lung sections. (**A**) Nuclear extraction and analyses of the H&E-stained image. Scale bar, 50 μm. (**B**) Box plot of the nuclear features extracted from the H&E-stained images of the normal lung (*n* = 40), benign (*n* = 4), and malignant (*n* = 36) areas from the resected specimens (Kruskal–Wallis test with Dunn’s multiple comparison test). ns, not significant. * *p* < 0.05, ** *p* < 0.01, *** *p* < 0.001, **** *p* < 0.0001.

**Figure 5:**
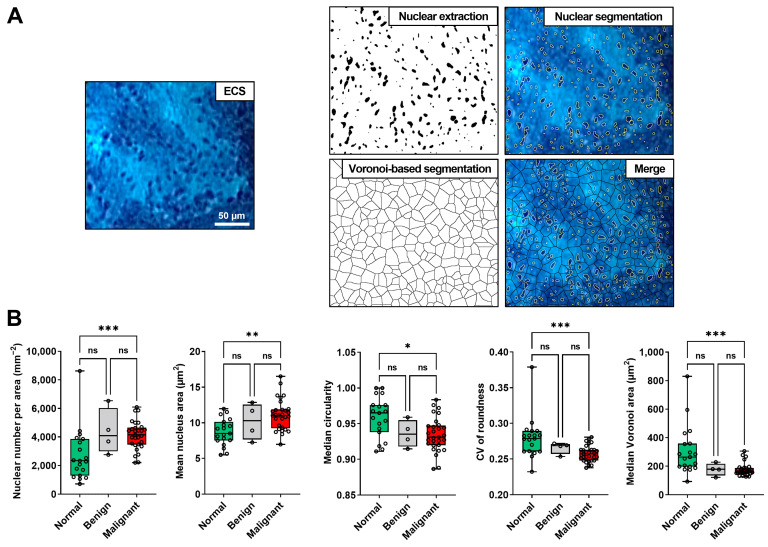
Nuclear feature extraction from the endocytoscopic images of methylene blue-stained resected lung specimens. (**A**) Nuclear extraction and analyses of the endocytoscopic image. Scale bar, 50 μm. (**B**) Box plot of nuclear features extracted from the endocytoscopic images of the normal lung (*n* = 19), benign (*n* = 4), and malignant (*n* = 29) areas from the resected specimens (Kruskal–Wallis test with Dunn’s multiple comparison test). ns, not significant. * *p* < 0.05, ** *p* < 0.01, *** *p* < 0.001.

To identify the features obtained from H&E-stained and endocytoscopic images, we determined the nuclear features of the normal lung, benign lesions, and malignant lesions via image analyses. All 40 lesions were included in the analysis of the H&E-stained images (Figure 2). Figure 4A depicts representative images of nuclear extraction and the quantification of nuclear features from an H&E-stained image of lung adenocarcinoma. Nuclear feature analysis revealed that the nuclear number per area and mean nucleus area were significantly higher in the malignant lesions than in the normal lung tissues (Figure 4B). Moreover, the median circularity, CV of roundness, and median Voronoi area were significantly higher in normal tissues than in malignant lesions. The nuclear number per area was also higher, and the median Voronoi area was lower in benign lesions than in normal lung tissues (Figure 4B). Similarly, we performed nuclear extraction and the quantification of the nuclear features from the endocytoscopic images (Figure 5A). Thirty-three (29 malignant and four benign lesions) of 40 lesions were included in the analysis of the endocytoscopic images, and 19 of 40 normal lung tissues were included in the analysis of the endocytoscopic images (Figure 2). Consistent with the H&E-stained image analysis, the mean nuclear number per area and mean nucleus area was significantly higher in the malignant lesions than in the normal lung tissues (Figure 5B). Moreover, the median circularity, CV of roundness, and median Voronoi area were significantly higher in the normal tissues than in the malignant lesions (Figure 5B). All five features were concordant between H&E-stained and endocytoscopic images. Value ranges of the normal lung tissues and malignant lesions were not thoroughly separated in each comparison.

### 3.4. Comparisons of Nuclear Features between H&E-Stained and Endocytoscopic Images

**Figure 6:**
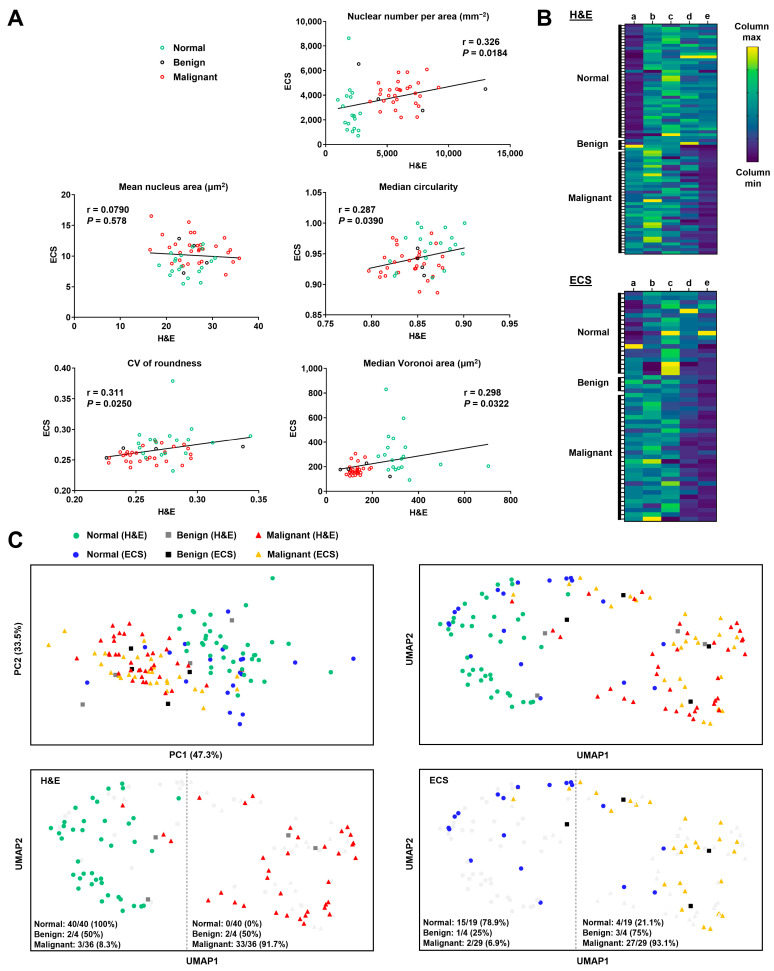
Comparison of the extracted nuclear features between the hematoxylin-eosin (H&E)-stained and endocytoscopic images in the resected lung specimens. (**A**) Linear regression analysis of each nuclear feature between the H&E-stained and endocytoscopic images (normal lung tissues, benign, and malignant lesions) using specimens of the same patients. CV, coefficient of variation. r, Pearson correlation coefficient (*n* = 52, simple linear regression). (**B**) Heatmaps of the min-max normalized nuclear feature values. Letters represent nuclear features: a, nuclear number per area; b, mean nucleus area; c, median circularity; d, CV of roundness; e, median Voronoi area. (**C**) Dimension reduction plots in nuclear features of the H&E-stained and endocytoscopic images using clustering embedding of principal component (PC) analysis (top-left) and uniform manifold approximation and projection (UMAP) clustering embedding with cosine distance as the metric.

We compared the values of H&E-stained and endocytoscopic nuclear features extracted from similar specimen areas of the same cases where an H&E-stained and endocytoscopic image were both obtained (malignant: 29 cases; benign: 4 cases; normal lung tissue: 19 cases) (Figure 6A). However, no feature values were correlated between the H&E-stained and endocytoscopic images. To obtain the data trend for all five nuclear features, we presented heat maps of the normalized nuclear feature values in the H&E-stained and endocytoscopic images (Figure 6B). Subsequently, we performed the PCA (Figure 6C, top-left) and UMAP (Figure 6C, top-right), respectively, using normalized values. PCA and UMAP clustering and embedding revealed analogous cluster locations between the H&E-stained images (malignant: 36 cases; benign: 4 cases; normal lung tissue: 40 cases) and endocytoscopic images (malignant: 29 cases; benign: 4 cases; normal lung tissue: 19 cases). We distinguished the normal lung-prone and malignant lesion-prone areas in the UMAP clusters (Figure 6C, H&E; bottom-left, ECS; bottom-right). UMAP clustering separated the normal and malignant clusters more than PCA did. Thus, the five nuclear features displayed similar trends in both H&E staining and ECS.

### 3.5. Inter-Observer Agreement in the Endocytoscopic Image Evaluation

**Figure 7:**
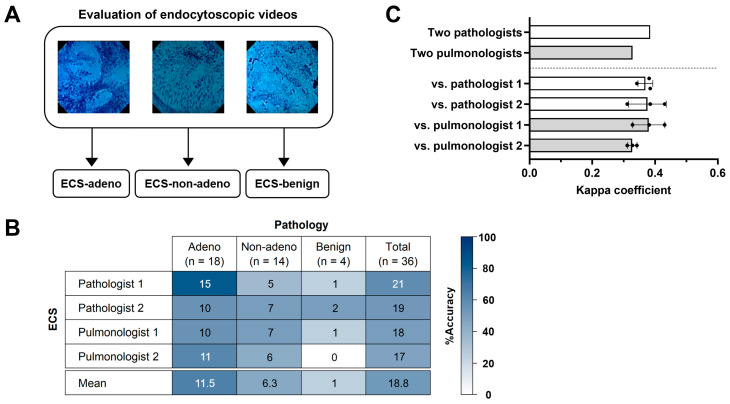
Evaluation of the endocytoscopic images of the methylene blue-stained resected lung specimens by two pathologists and two pulmonologists. (**A**) Schema of the evaluation of endocytoscopic images. (**B**) Diagnostic accuracy of the four observers compared with pathology. (**C**) The Kappa coefficient determining inter-observer agreement between each two of the four observers in the three categories (ECS-adeno, ECS-non-adeno, and ECS-benign).

To investigate agreements between observers of ECS, the pathologists and pulmonologists evaluated 36 cases from the recorded endocytoscopic videos (Figure 2 and Figure 7A). The typical lesions of “adenocarcinoma” and “squamous cell carcinoma” of the lungs obtained in this study (one of the 20 adenocarcinoma cases and one of the five squamous cases) were presented to each observer as labeled training videos before evaluation. Two of the labeled training videos were excluded from the evaluation.

We assessed the diagnostic accuracy (Figure 7B) and inter-observer agreement between the three categories (ECS-adeno, ECS-non-adeno, and ECS-benign) (Figure 7C). The total diagnostic accuracy of pathologists 1 and 2 was 58.3% (21/36) and 52.8% (19/36), respectively, and their inter-observer agreement was 0.38, i.e., fair. The total diagnostic accuracy of pulmonologists 1 and 2 was 50% (18/36) and 47.2% (17/36), respectively, and their inter-observer agreement was 0.33, i.e., fair. The diagnostic accuracy of each category (adeno, non-adeno, and benign) is also shown in Figure 7B. ECS-adeno was the best-matched category. Inter-observer agreement between each two of the four observers was between 0.31 and 0.43, i.e., fair or moderate.

## 4. Discussion

This study revealed that the five nuclear features (nuclear number per area, mean nucleus area, median circularity, CV of roundness, and median Voronoi area) significantly differed between the malignant lesions and normal lungs in both the H&E-stained and endocytoscopic images. Shibuya et al. reported that ECS during bronchoscopy could distinguish between normal bronchial mucosa, bronchial squamous dysplasia, and squamous cell carcinoma. They inserted the tip of the catheter-type endocytoscope through the biopsy channel of the bronchoscope and placed it on the bronchial surface, close to the site of interest. Endocytoscopic imaging of squamous cell carcinoma showed that cellular densities were increased and that the nuclear-cytoplasmic ratio was high and variable [11]. Shah et al. reported that ECS could distinguish normal epithelium from dysplasia and cancer [12]. Despite similarities between H&E-stained and endocytoscopic findings [7,8,15], they have not been quantitatively evaluated. To our knowledge, this is the first report of ECS in various types of pulmonary lesions and a quantitative investigation of endocytoscopic images.

We found that five nuclear features are beneficial for distinguishing between normal and malignant tissue in the resected specimens in this study. All five features were concordant between the H&E-stained and endocytoscopic images. The lower median Voronoi area may reflect the uneven distribution of malignant cells. In contrast, larger nucleus areas and lower median circularity were greatly related to the morphological changes of the malignant cell nuclei. Malignant lesions showed lower CV of roundness than normal lung tissue. The cells in normal lung tissue comprise various types, such as fibroblast, macrophages, and alveolar epithelial cells, and these nuclear shapes differ from one another. However, malignant lesions lose cell type diversity and have high cellular clonality. The nuclear size in the endocytoscopic images tended to be smaller than that in the H&E-stained images. The low-contrast endocytoscopic images with high background might provide less nuclear detection, thereby resulting in lower mean nuclear area and nuclear number per area than those of the existing nuclei.

Despite significant differences in the five nuclear features between the malignant lesions and normal lungs in the H&E-stained and endocytoscopic images, all five nuclear features were not correlated with the H&E-stained and endocytoscopic images in each feature of the same lesion. Wang et al. reported that the combinations of some extracted nuclear features from the H&E-stained images could predict recurrence in patients with lung cancer who underwent surgery [17]. Wolberg et al. presented 10 nuclear features obtained from breast cancer lesions useful in predicting whether the lesion was malignant or benign [18]. Similarly, in this study, the combination of all five nuclear features provided information on the malignant and normal lung cells since the UMAP-mapped data locations of the malignant lesions and normal lungs of the endocytoscopic images were considerably similar to those of the H&E-stained images. In clinical practice, pathologists determine the malignancy of a lesion based on the combination of multiple features of the cells and extracellular matrix. Our findings might also provide evidence for the importance of the five nuclear features in distinguishing lung malignancy.

In this study, we used H&E-stained images as the gold standard for comparison with the endocytoscopic images. We presented five representative cases with similar nuclear distribution and structures in both images, and ECS seems to be available for easily recognizing irregular nuclear distribution and piled-up nuclei on the alveolar wall. However, the in vivo usage of ECS in lungs poses some challenges owing to a thick-diameter bronchoscope, breathing movement, and DNA damage attributed to methylene blue staining [19]. Considering ex vivo application, ECS will likely be useful in determining whether the specimen of the pulmonary lesions is qualified for pathologic diagnosis during bronchoscopy. Evaluating the nuclear features of biopsy specimens using ex vivo ECS may reduce the number of biopsies by assessing the specimen’s amount and quality. In addition, ex vivo ECS may assist in the rapid pathologic diagnosis of tissues obtained by surgery, bronchoscopic biopsy, or CT-guided biopsy. This study is essential for developing endocytoscopic applications in the respiratory field.

In confocal laser endomicroscopy, Takemura et al. reported moderate inter-observer agreement among two pulmonologists who evaluated the cell density and nuclear size disparity of transbronchial biopsy specimens [20]. We examined the inter-observer agreement to investigate the diagnostic ability of ECS for surgically resected specimens. Although the pathologists are experts in pathological images, agreements were only fair between the pulmonologists and between the pathologists that carried out an ECS evaluation. As they have little experience observing endocytoscopic videos with only two training videos, training could improve the diagnostic ability of ECS. In addition, as a new approach in the gastroenterology field, crystal violet and methylene blue double staining [21,22] and artificial intelligence (AI) are used to improve the diagnostic ability of ECS [23]. Mori et al. reported on a computer-aided diagnosis using ECS and demonstrated a sensitivity and accuracy of 92.0% and 89.2%, respectively, for the classification of colorectal polyps [23]. AI plays an important role in pathology, such as the diagnosis of prostate cancer [24], breast cytology [18], and immunohistochemistry applications [25]. Moreover, AI may facilitate the on-site identification of histological types of biopsy specimens based on the nuclear features of endocytoscopic images.

This study has some limitations. First, several endocytoscopic images of normal lung areas were not used in the analyses because they were too dark. Second, we obtained only four cases of benign lesions owing to the low number of surgeries for benign diseases that included granuloma, lung cysts, and intrapulmonary lymph nodes. The features of benign lesions could not be shown.

In conclusion, the nuclear features of pulmonary lesions and normal lung tissue were similar in both the endocytoscopic and H&E-stained images. Endocytoscopic imaging could provide nuclear information similar to that of H&E-stained images.

## Data Availability

The data that support the findings of this study are available from the corresponding author, Noriaki Kurimoto, upon reasonable request.
